# Perspectives on rosacea patient characteristics and quality of life using baseline data from a phase 3 clinical study conducted in Japan

**DOI:** 10.1111/1346-8138.16596

**Published:** 2022-09-30

**Authors:** Kenshi Yamasaki, Yoshiki Miyachi

**Affiliations:** ^1^ Department of Dermatology Tohoku University Graduate School of Medicine Sendai Japan; ^2^ Rifu Dermatology & Allergology Clinic Rifu Japan; ^3^ Graduate School of Public Health Shizuoka Graduate University of Public Health Shizuoka Japan

**Keywords:** clinical trial, exacerbating factor, Japan, patient characteristics, quality of life, rosacea

## Abstract

There is a lack of contemporary data on rosacea originating in Japan. Using baseline data from a randomized, phase 3 study of 130 Japanese patients with rosacea treated with metronidazole gel (0.75%) or vehicle, the authors evaluated demographic and clinical characteristics, pretreatment quality of life, and exacerbating factors. In line with global data, most patients were women (82.3%; 107/130) and aged between 30 and 50 years (60.7%; 79/130). Patient‐reported quality of life scores indicated that rosacea had an impact similar to that of other debilitating and disfiguring skin conditions (such as psoriasis), particularly in terms of the emotional burden. Anxiety or depression was reported by 30% of patients (39/130), with 6.9% (9/130) reporting moderate levels and 0.8% (1/130) reporting severe levels. The top five exacerbating factors reported to trigger worsening of rosacea were temperature changes, sun exposure, hot weather, seasonal variation, and heavy exercise. In addition, pollen exposure and menstruation were noted as triggers of rosacea symptoms; these are novel findings that require further investigation to fully understand the implications for patients and treatment. Rosacea is likely to be underdiagnosed and undertreated in Japan because of the current lack of consensus guidelines and standardized therapy. The authors anticipate that the results of this analysis will provide much needed information to help improve diagnosis and facilitate the management of rosacea in patients.

## 1 INTRODUCTION

Rosacea, a chronic inflammatory dermatologic disorder, is characterized by flushing or erythema, papules, pustules, and telangiectasia.^1^ It mainly affects the cheeks, nose, chin, and forehead, and occasionally produces ocular manifestations and involves other bodily locations.^2^ The global prevalence of rosacea has been estimated at 5.5% of the adult population,^3^ although most information on rosacea comes from White patients in Western countries.^4^ In contrast to the many publications relating to rosacea from Europe and the United States, there is a lack of contemporary data originating in Japan. The most recent report of Japanese rosacea prevalence was published a decade ago; in that cross‐sectional study, the authors found that a rosacea diagnosis was made in 0.22% of 67 448 patients who visited dermatology clinics in Japan in May, August, or November 2007 or in February 2008.^5^ Among the data available for other Asian countries, prevalence rates vary widely, from a few percent up to as high as 20%.^4^, ^6^ The low estimation of Japanese rosacea prevalence is likely attributable to a lack of consensus in how rosacea is identified and measured and possibly also to a biased expectation of a low prevalence of rosacea by Japanese physicians.

Based on data from Western populations, it is generally believed that adults aged between 30 and 50 years, women, and individuals with fair skin are most likely to develop rosacea.^1^, ^7^ Conversely, symptoms may be more severe in men and in younger patients.^8^ The persistent facial erythema associated with rosacea is reported to negatively impact quality of life (QoL).^9^ According to data obtained from 600 participants who completed a web‐based survey in the United States, up to 90% of individuals with rosacea reported experiencing diminished self‐esteem and self‐confidence, and 41% reported avoiding social activities because of the effect of rosacea on their appearance.^10^ While the QoL burden on Japanese patients with rosacea is expected to be similar, confirmatory data have been lacking.

Many factors, such as exposure to UV radiation, local inflammatory responses to skin microorganisms, temperature changes, spicy foods and alcohol, heavy exercise, and stress have been implicated in the initiation or worsening of rosacea.^11^, ^12^ Several European and North American medical societies and expert consensus panels have published independent recommendations and advocate skin care and the avoidance of exacerbating factors.^13^, ^14^, ^15^, ^16^, ^17^ The Japanese Dermatological Association published guidelines for the treatment of acne vulgaris in 2017, in which rosacea was mentioned and general skin care (protection from UV radiation, low‐irritant cleansing, and moisturization) was advised.^18^ In order to achieve the best outcomes for Japanese patients, it is first necessary to understand the current parameters of the disease within the Japanese population with rosacea. Although a recent single‐center, retrospective analysis reported on the background characteristics of 340 Japanese patients with rosacea,^19^ prospectively collected data from across Japan are still needed as a basis from which to develop national guidelines and treatment recommendations.

We have reported the results from a randomized, vehicle‐controlled, phase 3 study of metronidazole gel (0.75%) in the treatment of Japanese patients with rosacea, which was conducted from April 2019 to May 2020.^20^ In the trial, patients with rosacea were enrolled earlier than expected, suggesting that the true number of affected patients in Japan is higher than previous reports would indicate and supporting the need for accurate diagnostic criteria. In the current analysis, using the clinical and demographic data collected during the phase 3 study, we evaluated the baseline characteristics, pretreatment QoL, and exacerbating factors of Japanese patients with rosacea, with the aim of better understanding the landscape of rosacea in Japan. To our knowledge, this is the first published report to prospectively compile background information on a large population of Japanese patients with rosacea.

## 2 METHODS

### 2.1 Patients

Details of the patients included in the phase 3 study (registered with the Japan Pharmaceutical Information Center Clinical Trials Information registry as JapicCTI‐194 688) have been reported.^20^ The key inclusion criteria at baseline were age ≥ 18 years, a moderate Investigator Global Assessment (IGA) score (≥3), an inflammatory lesion count (papules/pustules) on the whole face of ≥11 and ≤ 40, and a mild erythema severity score (≥2).

All patients and their legal guardians provided written informed consent for study participation. The multicenter study (26 sites) was conducted between April 2019 and May 2020, and all procedures were performed in compliance with the Declaration of Helsinki, good clinical practice, and all relevant legislation. The study protocol was reviewed and approved by the institutional review board at each participating site.^20^

### 2.2 Items evaluated

The clinical rosacea status was assessed by the investigator, based on the number of inflammatory lesions (papules/pustules), erythema severity, and the IGA. Erythema severity was scored on a five‐point scale from 0 (no erythema) to 4 (severe erythema), and the IGA score was also based on a five‐point scale from 0 (clear; no inflammatory lesion and no erythema) to 4 (severe; many small to large papules and pustules or severe erythema).

Patient‐reported QoL measures included the Dermatology Life Quality Index (DLQI),^21^ the Skindex‐16,^22^ and the EuroQol 5‐dimension 5‐level (EQ‐5D‐5L) questionnaire.^23^ The 10‐item DLQI yields a score from 0 to 30, and the 16‐item Skindex‐16 scores recent symptoms on a scale from 0 (never bothered) to 6 (always bothered); for both measures, higher scores indicate a greater disease impact. The EQ‐5D‐5L comprises five dimensions (mobility, self‐care, usual activities, pain/discomfort, and anxiety/depression) scored on five levels (no problems to extreme problems) plus a visual analog scale (VAS) of health status.

### 2.3 Statistical analyses

For this exploratory analysis, no formal statistical calculations were performed. Data were recorded descriptively using number (percentage) for categorical variables and mean (standard deviation [SD]) for continuous variables. All analyses were performed using SAS version 9.4 (SAS Institute Inc.).

## 3 RESULTS

A total of 130 patients met the study criteria and were assigned to study treatment; data from all 130 patients were used to explore demographic and clinical characteristics, exacerbating factors, and pretreatment QoL in this analysis.

### 3.1 Baseline characteristics

Demographic and clinical variables are summarized in Table 1. Men comprised 17.7% of the population (23/130). Many patients were aged between 40 and 50 years (41.5%; 54/130), with a mean age at onset of rosacea of 43.8 (SD, 14.3) years. The mean duration of disease was 4.7 (SD, 6.8) years. Seasonal allergy was identified in more than a quarter of patients (26.9%; 35/130). Additional associated medical conditions that were each reported in >5% of patients were hypertension, constipation, hyperuricemia, insomnia, migraine, allergic rhinitis, and urticaria.

**TABLE 1:** Baseline demographic and clinical characteristics of patients with rosacea

Variable	Phase 3 full analysis set (*N* = 130)
Men, *n* (%)	23 (17.7)
Age, years	
Range, *n* (%)	
<20	0
≧20 to <30	6 (4.6)
≧30 to<40	25 (19.2)
≧40 to <50	54 (41.5)
≧50 to <60	23 (17.7)
≧60 to <70	12 (9.2)
≧70 to <80	7 (5.4)
≧80	3 (2.3)
Mean (SD)	47.8 (13.0)
Rosacea timeline, years, mean (SD)^a^	
Age of onset	43.8 (14.3)
Duration	4.7 (6.8)
Associated medical conditions in >5% of patients, *n* (%)	
Seasonal allergy (pollen)	35 (26.9)
Hypertension	15 (11.5)
Constipation	9 (6.9)
Hyperuricemia	9 (6.9)
Insomnia	9 (6.9)
Migraine	9 (6.9)
Allergic rhinitis	8 (6.2)
Urticaria	8 (6.2)

Abbreviation: SD, standard deviation.

^a^ Data were available for 121 of 130 patients.

### 3.2 Investigator‐assessed rosacea severity

The mean number of baseline inflammatory lesions including papules and pustules was 23.7 (SD, 9.3). Patients tended to have more papules than pustules. Many patients had an erythema severity score of 3 (60.8%; 79/130), and most had an IGA score of 3 (87.7%; 114/130; Table 2).

**TABLE 2:** Summary of investigator‐assessed measures for the symptoms and severity of rosacea

Measures	Phase 3 full analysis set (*N* = 130)
Inflammatory lesions (papules + pustules)	
Range, *n* (%)	
11–20	64 (49.2)
21–30	28 (21.5)
31–40	38 (29.2)
Number, mean (SD)	23.7 (9.3)
Number of papules, mean (SD)	21.1 (8.6)
Number of pustules, mean (SD)	2.7 (3.9)
Erythema severity, *n* (%)	
Mild (score 2)	28 (21.5)
Moderate (score 3)	79 (60.8)
Severe (score 4)	23 (17.7)
IGA, *n* (%)	
Moderate (score 3)	114 (87.7)
Severe (score 4)	16 (12.3)

Abbreviations: IGA, Investigator Global Assessment; SD, standard deviation.

### 3.3 Patient‐reported QoL

QoL scores at baseline are summarized in Table 3. The mean DLQI total score was 3.9 (SD, 3.6) and the Skindex‐16 overall score was 35.4 (SD, 22.9). For the EQ‐5D‐5L, the mean utility and VAS mean scores were 0.901 (SD, 0.112) and 76.6 (SD, 15.5), respectively. In terms of the EQ‐5D‐5L individual items, just under half of patients reported pain or discomfort resulting from their rosacea, with 5.4% (7/130) reporting moderate discomfort and 3.8% (5/130) reporting severe discomfort. Anxiety or depression was reported by 30% of patients (39/130), with 6.9% (9/130) reporting moderate levels and 0.8% (1/130) reporting severe levels.

**TABLE 3:** Summary of patient‐reported quality of life measures

Measures	Phase 3 full analysis set (*N* = 130)
DLQI, mean (SD)	
Total score	3.9 (3.6)
Symptoms and feelings^a^	2.0 (1.3)
Daily activities^a^	0.6 (1.0)
Leisure^a^	0.7 (1.1)
Work and school^b^	0.3 (0.7)
Personal relationships^a^	0.1 (0.4)
Treatment^b^	0.2 (0.5)
Skindex‐16, mean (SD)	
Overall score	35.4 (22.9)
Symptoms	25.9 (26.9)
Emotions	60.2 (30.4)
Functioning	20.0 (25.4)
EQ‐5D‐5L	
Level of pain/discomfort, *n* (%)	
None	71 (54.6)
Slight	47 (36.2)
Moderate	7 (5.4)
Severe	5 (3.8)
Extreme	0
Level of anxiety/depression, *n* (%)	
None	91 (70.0)
Slight	29 (22.3)
Moderate	9 (6.9)
Severe	1 (0.8)
Extreme	0
Utility value, mean (SD)	0.901 (0.112)
VAS score, mean (SD)	76.6 (15.5)

Abbreviations: DLQI, Dermatology Life Quality Index; EQ‐5D‐5L, EuroQol 5‐dimension 5‐level; QoL, quality of life; SD, standard deviation; VAS, visual analog scale.

^a^ Maximum score = 6.

^b^ Maximum score = 3.

### 3.4 Factors worsening rosacea symptoms

Exacerbating factors reported to trigger worsening of rosacea are shown in Table 4. Overall, seasonal factors, including temperature, sun exposure, heat/cold, and pollen exposure, were frequently cited as triggers. Other common exacerbating factors were exercise, emotional stress, and food/alcohol intake. Notably, 7.7% (10/130) of all patients reported no exacerbating factors. Some differences in exacerbating factors according to sex were also observed, including menstruation and cosmetic use (more common in women) and intake of spicy foods and alcohol (more common in men).

**TABLE 4:** Exacerbating factors associated with worsening rosacea, overall and by sex

Exacerbating factor, *n* (%)	Phase 3 full analysis set (*N* = 130)	Men (*n* = 23)	Women (*n* = 107)
Temperature changes	70 (53.8)	11 (47.8)	59 (55.1)
Sun exposure	51 (39.2)	10 (43.5)	41 (38.3)
Hot weather	46 (35.4)	6 (26.1)	40 (37.4)
Seasonal variation	39 (30.0)	5 (21.7)	34 (31.8)
Heavy exercise	36 (27.7)	4 (17.4)	32 (29.9)
Emotional stress	32 (24.6)	5 (21.7)	27 (25.2)
Pollen	31 (23.8)	1 (4.3)	30 (28.0)
Alcohol	30 (23.1)	8 (34.8)	22 (20.6)
Menstrual cycle	25 (19.2)	0	25 (23.4)
Cold weather	24 (18.5)	3 (13.0)	21 (19.6)
Hot (temperature) foods	22 (16.9)	6 (26.1)	16 (15.0)
Use of cosmetics	16 (12.3)	0	16 (15.0)
Spicy foods	14 (10.8)	5 (21.7)	9 (8.4)
Others	15 (11.5)	2(8.7)	13(12.1)
None	10 (7.7)	3 (13.0)	7 (6.5)
None of the top five factors^a^	34 (26.2)	8 (34.8)	26 (24.3)

*Note*: More than one exacerbating factor could be selected per patient.

^a^ Temperature changes, sun exposure, hot weather, seasonal variation, heavy exercise.

## 4 DISCUSSION

In the current study we analyzed the baseline data from a phase 3 clinical trial of Japanese patients with rosacea, collated from multiple dermatology clinics and hospitals throughout Japan, as a source of contemporary information for Japanese clinicians. Prior reports in countries outside of Japan have suggested that rosacea tends to occur in patients aged between 30 and 50 years.^1^, ^7^ Our data would appear to support this trend, with the majority of study patients (60.7%) aged between 30 and 50 years, and a mean age of rosacea onset of 43.8 years. Prior epidemiologic reports have suggested that approximately three times more women than men are affected by rosacea,^1^, ^24^ and in our study, men comprised just 17.7% of the population.

Any condition leading to facial skin signs can cause the loss of self‐esteem, increase self‐consciousness and embarrassment, and negatively impact willingness to interact with others in a social or work environment.^25^ A meta‐analysis of data from seven studies found that patients with rosacea were more likely to assess their symptoms as severe, compared with clinicians; this self‐assessment is likely to drive the elevated rates of anxiety/depression, self‐consciousness, frustration, and interference with daily life reported by patients.^26^ In the current analysis, all three measures used (DLQI, Skindex‐16, and EQ‐5D‐5L) indicated reduced QoL for Japanese patients with rosacea compared with healthy individuals, with particularly poor scores in the areas of emotional well‐being and levels of anxiety/depression. In normal populations, mean DLQI scores range from 0–0.5.^27^ Prior evaluations of the DLQI in patients with rosacea have reported total scores ranging from 4.3 to 17.3,^28^ which are consistent with our data. Similar correlations between self‐reported illness perception and QoL have been demonstrated in other dermatoses, such as acne, psoriasis, and eczema,^29^ bullous pemphigoid,^27^ and vitiligo.^30^ Psoriasis has been shown to have a greater impact on QoL than angina and hypertension,^28^ while acne produces QoL deficits comparable to those observed in patients with asthma, epilepsy, diabetes, or arthritis.^31^ Mean Skindex‐16 scores in the current study were consistent with a prior study in Japanese patients with psoriasis.^22^ Of note, the emotional burden of rosacea measured by the Skindex‐16 was particularly high in the current study (mean score 60.2), reflecting the worry and self‐consciousness patients feel about their condition. A similarly high emotional burden has been reported for patients with hidradenitis suppurativa (acne inversa).^32^ The EQ‐5D‐5L scores in the rosacea population in this study were also largely driven by rates of pain/discomfort and anxiety/depression, further supporting the need to treat both physical symptoms and mental distress in affected patients.^33^

While patient demographic characteristics were aligned between the Japanese patients in our study and non‐Japanese patients in prior publications, exacerbating factors were found to be different. A survey of 1066 patients with rosacea conducted by the National Rosacea Society (NRS) in the United States reported that sun exposure was the most frequently reported exacerbating factor (81%), followed by emotional stress (79%) and hot weather (75%) (Table S1).^34^, ^35^ In contrast, the most frequently reported exacerbating factor in the current study was temperature changes (53.8%). Interestingly, the recent retrospective study conducted at Tohoku University also reported that temperature differences were the most frequent external exacerbating factor for rosacea.^19^ Conversely, sun exposure was reported as an exacerbating factor by only 39.2% of Japanese patients in our study compared with 81% in the US NRS study. Since differences in exacerbating factors according to degree of skin pigmentation have been reported,^36^, ^37^ it appears likely that skin type or pigmentation may influence the degree of importance of specific exacerbating factors in Japan. Although these findings require corroboration in large‐scale studies, differences in key exacerbating factors between Western and Japanese populations may need to be taken into account by dermatologists when they diagnose and treat patients with rosacea.

Interestingly, pollen was reported as an exacerbating factor by a quarter of Japanese patients (23.8%), but was not mentioned in the US study.^34^, ^35^ Patients with rosacea have a significantly greater likelihood of sensitivity to airborne and other allergens,^38^ and a dysfunctional immune response has been implicated in the development of rosacea.^39^ Among patients with rosacea in the Tohoku University study who received allergy tests, 59% were found to have IgE specific to the pollen of Japanese cedar trees, and a further 20% of patients had allergies to the pollen of Japanese cypress, orchard grass, and timothy grass.^19^ Although our study did not address individual pollen types, to our knowledge, it is the first report of pollen as a direct trigger for rosacea worsening, and further investigation will be needed to determine whether this is a factor specific to Japanese or Asian patients. The coexistence of rosacea and pollen allergies has implications for treatment since rosacea symptoms may not fully improve unless the complicating allergic condition is also addressed.

Another factor mentioned as an exacerbating factor by 19.2% of Japanese patients, but not raised in the NRS survey, was the menstrual cycle. However, recently published data from the Nurses Health Study II in White US women indicated that hormonal and reproductive factors may increase the risk of rosacea, likely via the effects of sex hormones on the immune response.^40^ Another study, in Korean patients with rosacea, found that menstruation was an aggravating factor only for the neurogenic type of rosacea and not the erythematotelangiectatic type.^41^ More data are needed to obtain a better insight into the temporal pattern of rosacea worsening during the menstrual cycle and the underlying pathophysiology. Additional exacerbating factors with an underlying sex‐related difference included the use of cosmetics (women only), and lifestyle factors such as intake of spicy foods and alcohol (more common in men). Further research is necessary to understand fully the spectrum of rosacea triggers, and we suggest that clinicians and researchers widen their queries for patients with rosacea beyond the standard exacerbating factors of season, weather, and temperature.

Notably, patient enrollment into the phase 3 clinical study was completed rapidly, in a shorter period than anticipated, potentially suggesting a higher prevalence of rosacea in Japan than expected by dermatologists. Given that rosacea is likely underdiagnosed as a result of a lack of consensus guidelines in Japan, the true prevalence of rosacea would appear to be much higher than the reported rates.^5^ In real‐world clinical practice, the implementation of accurate rosacea criteria and careful differential diagnosis would improve rates of diagnosis and reduce misdiagnosis of this condition. Because of the diverse presentations of rosacea, it must first be differentiated from other skin conditions with overlapping manifestations,^42^ and the approaches to treatment must be individualized based on the disease severity, QoL implications, comorbidities, exacerbating factors, and the patient's commitment to therapy. By combining the NRS standard rosacea classifications^43^ with the simple physical observations and medical history used to confirm the diagnosis in the phase 3 study (summarized in Table S2), physicians in Japan will be able to reduce both the time to arrive at an accurate diagnosis and the time to initiate proper treatment.

Despite the burden placed on individuals with rosacea in Japan, treatment options are extremely limited. While there are independent guidelines specific to rosacea management in Europe and North America,^13^, ^14^, ^15^, ^16^, ^17^ none exist in Japan. Insurance coverage for pharmacologic treatment for rosacea is virtually nonexistent. Japanese physicians are advised to manage symptoms using general skin care regimens,^18^ but skin care alone may prove insufficient for many patients.^44^ In contrast, multiple treatments have been approved for use by the US Food and Drug Administration, including ivermectin 1% cream, metronidazole 0.75% cream, metronidazole gel 1%, and azelaic acid 15% gel or foam for inflammatory lesions, and brimonidine gel 0.33% and oxymetazoline cream 1% for erythema.^45^, ^46^ It seems clear that consensus guidelines for treatment as well as diagnosis of rosacea in Japan are needed, particularly in light of recent research into the use of potentially beneficial therapies such as metronidazole gel (0.75%).^20^

Limitations of the current analysis include its exploratory nature, via the use of data collected during the phase 3 clinical study. Because of the inclusion and exclusion criteria implemented during the study, the patient population may not be entirely representative of all Japanese patients with rosacea; specifically, we evaluated only patients who were determined to have at least mild erythema severity and a moderate IGA score. In addition, the current analysis only included patients aged ≥18 years, as the number of affected children and adolescents in Japan is thought to be small. However, given the current paucity of data relating to the characteristics of patients with rosacea in Japan, we nonetheless consider that the information obtained are broadly applicable to the general clinical population with rosacea in Japan, and will serve as a basis to enable clinicians to improve and optimize the diagnosis and management of these patients over the coming years.

## 5 CONCLUSIONS

For Japanese patients with rosacea, the lack of consensus guidelines and standard treatment has resulted in an underestimation of the scale of the problem and a paucity of management options. Our data have elucidated the key exacerbating factors for rosacea in the Japanese population and highlighted potential areas of difference from Western populations deserving of additional research. We have also shown that patients with rosacea have an impaired quality of life, driven by both physical symptoms and emotional distress, placing a heavy burden on those affected by this condition. Improvements in diagnostic accuracy and the formulation of consensus Japanese guidelines for both diagnosis and treatment are needed to advance the differential diagnosis of rosacea and lessen the current unmet clinical need.

## CONFLICT OF INTEREST

This study was funded by Maruho. K.Y. and Y.M. have received research funding, consultancy fees, speaker fees, fees for arranging education, and personal fees from Maruho.

## Supporting information


Tables S1‐S2
