# Supplementary material for: Perspectives on rosacea patient characteristics and quality of life using baseline data from a phase 3 clinical study conducted in Japan
Source: J Dermatol. 2022 Sep 30;49(12):1221–7. doi: 10.1111/1346-8138.16596 (PMC10092295; doi:10.1111/1346-8138.16596)
Supplement: Supplementary file 1 — Tables S1‐S2 [file JDE-49-1221-s001.docx]

**Supplementary data**

**Table S1. Top 10 most frequent rosacea triggers from the National Rosacea Society survey of 1,066 rosacea patients**[**^34^**](#_ENREF_34)**^,^** [**^35^**](#_ENREF_35)

| **Factors** | **Patients affected, %** |
| --- | --- |
| Sun exposure | 81.0 |
| Emotional stress | 79.0 |
| Hot weather | 75.0 |
| Wind | 57.0 |
| Heavy exercise | 56.0 |
| Alcohol consumption | 52.0 |
| Hot baths | 51.0 |
| Cold weather | 46.0 |
| Spicy foods | 45.0 |
| Humidity | 44.0 |

Table reproduced from Johnson SM, Berg A, Barr C. Managing rosacea in the clinic: From pathophysiology to treatment. A review of the literature. *J Clin Aesthet Dermatol*. 2020;13(4 Suppl):S17-S22, and reprinted here with the permission of the publisher.

**Table S2. Differential diagnosis of rosacea**

| **Condition** | **Diagnostic details** |
| --- | --- |
| Rosacea[^18^](#_ENREF_18)^,^ [^43^](#_ENREF_43) | - Main symptoms: transient or persistent erythema, papules and pustules, telangiectases   - Erythema is diffuse (including the cheeks, nose tip, glabella, and chin)   - No comedones; papules and pustules are not always pore-matched - Secondary symptoms: burning or stinging of the skin, red plaque, dry appearance, edema, ocular symptoms, peripheral manifestations, rhinophyma - Appears mainly on the face in middle age - Unknown cause and symptoms persist for more than a few months |
| Acne[^47^](#_ENREF_47) | - Main symptoms: papules, pustules, erythema; however, the presence of comedones is a criterion for diagnosis   - No telangiectasia or hot flashes   - No diffuse erythema - Commonly found on the face, but also on the back, and on the central part of the chest; generally appears around puberty - Most cases improve with adapalene gel treatment, whereas rosacea does not |
| Rosacea-like dermatitis[^48-51^](#_ENREF_48) | - Main symptoms: papules or pustules on an erythematous base, usually localized in seborrheic areas; may be described as acneiform - Commonly occurs following long-term treatment with topical steroids or associated with systemic immunotherapies; symptoms are generally dose-dependent, transient, and reversible - When the symptoms of rosacea continue for more than 3 months after discontinuation of topical steroids, patients can be diagnosed with rosacea rather than with a complication of rosacea-like dermatitis |
| Demodex folliculorum^[52-55](#_ENREF_52)^ | - Main symptoms: rough skin, scaly or itchy skin, erythema or rash, burning feeling, eczema, blepharitis   - Mites are found in seborrheic regions including facial sebaceous glands, but also in peri-orbital areas, the genital region, and the buccal mucosa - Patients with rosacea are more likely to have *Demodex* infestation compared with healthy controls, but not all patients with *Demodex* mites have rosacea - Even if *Demodex folliculorum* is detected, if it is accompanied by irregular telangiectasia or transient flushing, it can be diagnosed as rosacea rather than as a complication of *Demodex folliculorum* |
| Seborrheic dermatitis[^56^](#_ENREF_56) | - Main symptoms: red patches and scabs with white greasy scale; in those with darker skin tones, petaloid or hypo-pigmented plaques may be observed - Affects the scalp and face, particularly the nasolabial folds, eyebrows, ears, post-auricular areas, and (in men) in the areas of the beard and chest hair - Occurs at all ages, including infants, adolescents, and adults - Post-inflammatory alterations may remain after seborrheic dermatitis has been cured; patients who have previously been diagnosed with seborrheic dermatitis may subsequently be diagnosed with rosacea |
